# Atmospheric Deposition of Nitrogen and Sulphur Compounds in the Czech Republic

**DOI:** 10.1100/tsw.2001.88

**Published:** 2001-09-24

**Authors:** Milos Zapletal

**Affiliations:** Centre for Environment and Land Assessment-Ekotoxa Opava, Czech Republic

**Keywords:** atmospheric deposition, acid deposition, nitrogen oxides, ammonia/ammonium, sulphur oxides, dry deposition, wet deposition, deposition velocity, resistance model

## Abstract

Estimates of dry and wet deposition of nitrogen and sulphur compounds in the Czech Republic for the years 1994 and 1998 are presented. Deposition has been estimated from monitored and modeled concentrations in the atmosphere and in precipitation, where the most important acidifying compounds are sulphur diO_x_ide, nitrogen O_x_ides, ammonia, and their reaction products. Measured atmospheric concentrations of SO_2_, NO_x_, NH_3_, and aerosol particles (SO_4_, NO_3_, and NH_4_), along with measured concentrations of SO_4_, NO_3_, and NH_4_ in precipitation, weighted by precipitation amounts, were interpolated with Kriging technique on a 10- x 10-km grid covering the whole Czech Republic. Wet deposition was derived from concentration values for SO_4_, NO_3_, and NH_4_ in precipitation and from precipitation amounts. Dry deposition was derived from concentrations of gaseous components and aerosol in the air, and from their deposition velocities. A multiple resistance model was used for calculation of SO_2_, NO_x_, and NH_3_ deposition velocities. Deposition velocities of particles were parameterized. It was estimated that the annual average deposition of SO_x_ in the Czech Republic decreased from 1384 to 1027 mol H ha a between 1994 and 1998. The annual average NO_y_ deposition was estimated to be 972 and 919 mol H a in 1994 and 1998, respectively. The annual average NH_x_ deposition was estimated to be 887 mol H a and 779 mol H a in 1994 and 1998, respectively. It was estimated that the annual average of the total potential acid deposition decreased from 3243 to 2725 mol H a between 1994 and 1998. Sulphur compounds (SO_x_) contributed about 38%, O_x_idized nitrogen species (NO_y_) 34%, and reduced nitrogen species (NH_x_) 28% to the total potential acid deposition in 1998. The wet deposition contributed 42% to the total potential acid deposition in 1998.

### INTRODUCTION

The environment in the Czech Republic showed an increasing damage to forests due to acid deposition. For the assessment of potential effects of air pollution on ecosystems, it is essential to know the actual atmospheric deposition load. It is necessary to know where threshold deposition loads are exceeded and which compounds contribute most to the loads. In this way abatement measures on emission controls can be optimized. The working plan for the implementation of the UN ECE Convention on Long-range Transboundary Air Pollution (LRTAP) includes the production of maps of deposition loads, critical loads, and exceedances as a basis for developing potential abatement strategies for nitrogen and sulphur. The aim of this study is to estimate deposition fluxes of nitrogen and sulphur compounds on a 10- × 10-km scale in the Czech Republic for the period from 1994 to 1998. This study summarizes some important results from the Czech Programme on Acidification[1,2,3,4,5,6,7,8], which was carried out on behalf of the Ministry of the Environment of the Czech Republic from 1993 to 2000.

## EXPERIMENTAL METHODS/PROCEDURES

### Acid Atmospheric Deposition

The following acidifying components were considered in this study: sulphur dioxide ($SO_2$), nitrogen oxides (NO, $NO_2$), nitric acid ($HNO_3$), ammonia ($NH_3$), sulphates ($SO_4^{2-}$), nitrate ($NO_3^-$), and ammonium ($NH_4^+$) in aerosol, in air, and in precipitation, respectively. One mole of $SO_2$ can form two equivalents of acid; one mole of $NO_x$ or $NH_3$ can form one equivalent of acid[9]. The maximum amount of acidifying components removed from the atmosphere by deposition, hereafter referred to as total potential acid deposition, is estimated by

$$\text{Total potential acid} = 2\ SO_x + NO_y + NH_x \qquad (1)$$

where $SO_x$ is the total of oxidized sulphur compounds (gaseous $SO_2$ and $SO_4^{2-}$ particulates in air and precipitation), where $NO_y$ is the total deposition of oxidized nitrogen compounds (NO, $NO_2$, $HNO_3$, and $NO_3^-$ in air and precipitation), and where $NH_x$ is the total deposition of reduced nitrogen compounds ($NH_3$ and $NH_4^+$ in air and precipitation).

### Wet Deposition

For the purpose of this study, the concentrations of $SO_4^{2-}$, $NO_3^-$, and $NH_4^+$ in precipitation, collected by monitoring stations of the Czech Hydrometeorological Institute[10,11], the Water Management Research Institute T.G.M.[4,8], and the Czech Geological Survey[4,8], were available. Precipitation at most of these stations was collected by the bulk method (samplers are exposed continuously, and are therefore enriched with dry deposition). At a few stations of the Czech Hydrometeorological Institute, the samples were collected by the so-called wet-only method (an automatic collection of precipitations excluding the contribution of dry deposition). Correction factors were applied to correct the dry deposition in precipitation collected by the bulk method. The concentrations of $SO_4^{2-}$, $NO_3^-$, and $NH_4^+$ in precipitation collected by the bulk method were estimated to be higher than those in precipitation collected by the wet-only method. This was done on the basis of comparison of parallel measurements, performed both by the bulk and wet-only method, consideration of geographic location of monitoring stations (clear or polluted areas), frequency of precipitation collection, and the average of correction factors presented in the literature[5]. In a medium the difference for $SO_4^{2-}$ was 26%, for $NO_3^-$ 20%, and for $NH_4^+$ 29%.

The total potential acid wet deposition of nitrogen and sulphur compounds estimated in this study is

$$\text{Total potential acid (wet deposition)} = 2\ SO_4^{2-} + NO_3^- + NH_4^+ \qquad (2)$$

Annual average wet deposition of $SO_4^{2-}$, $NO_3^-$, and $NH_4^+$ for every 10- × 10-km grid on the territory of the Czech Republic in 1994 and 1998 was computed as a product of the annual average concentrations of $SO_4^{2-}$, $NO_3^-$, and $NH_4^+$ in precipitation and of the annual precipitation amounts.

### Dry Deposition

Dry deposition fluxes were estimated from measured concentrations of gases and aerosol particles in air multiplied by the corresponding deposition velocities:

$$F = V_d(z)C(z) \qquad (3)$$

where $F$ is the deposition flux of the component to a unit area (e.g., $m^2$, ha), $V_d$ is the deposition velocity of the component, and $C(z)$ is the concentration of the component at a height $z$ above surface.

The concentrations of $SO_2$, nitrogen oxides ($NO_x$), and dust aerosol are routinely monitored and evaluated in the Czech Republic. For this study the databases of the Air Quality Information System of the Czech Republic [10,11] and the Ekotoxa Opava[4,8] were used. These two monitoring networks consist partly of manually controlled stations on a 24-h basis, using absorption–photometric measuring methods (286 stations), partly of automatic monitoring stations operating continuously on a 30-minute time resolution (132 stations). Annual average concentrations of $NO_x$ and $SO_2$ computed from daily measurements were interpolated using Kriging technique on the 10- × 10-km resolution grid. Annual average concentrations of $NH_3$, $HNO_3$, $SO_4^{2-}$, $NO_3^-$, and $NH_4^+$ on the territory of the Czech Republic were not available for the years 1994 and 1998, therefore the data from the EMEP-LRTAP model[12] at 150- × 150-km resolution for 1993 had been used. Annual average concentrations of $NH_3$ in 1993 from the EMEP-LRTAP model were corrected according to a spatial distribution of the annual emissions of $NH_3$ on a 10- × 10-km grid for the Czech Republic territory for the years 1994 [4] and 1998[8]. Annual emissions of $NH_3$ in 1994 and 1998 were computed by averages of the emission model on the basis of precise total emission balance of $NH_3$ from all sources (livestock, manures, artificial fertilizers, natural losses of soil, humans, wastes, forest soils, and industry) for the years 1994 and 1998[4,5,8]. For the other components ($HNO_3$ and aerosol), the annual average concentration was extrapolated onto a 10- × 10-km grid.

Deposition velocity for gases was calculated using the resistance analogy[13,14]. Deposition velocity, $V_d$, may be expressed by the inverse of the sum of three resistances:

$$V_d(z) = \frac{1}{R_a(z) + R_b + R_c} \qquad (4)$$

The three resistances represent three stages of transport: the aerodynamic resistance, $R_a$, for the turbulent layer, the laminar layer resistance, $R_b$, for the quasi-laminar layer, the surface or canopy resistance, $R_c$, for the receptor itself. In this study the aerodynamic resistance, $R_a$, is calculated from micrometeorological relations suggested by Voldner et al. and Hicks et al.[13,15], and the quasi-laminar layer resistance, $R_b$, is calculated from micrometeorological relations suggested by Hicks et al.[15]. $R_a$ and $R_b$ may be assessed on the basis of known wind velocity and surface roughness. The annual average values of surface roughness, $z_0$, for different surface types were derived from the literature[13,14]. Annual averages of the surface roughness, $z_0$, were related to the corresponding surface characteristics on the territory of the Czech

Republic according to the geographical model of the land-use types at 1- × 1-km resolution[1]. The land-use classes used are coniferous forests, deciduous forests, cultivated land, grassland, urban areas, and water surfaces, e.g., lakes and rivers.

Surface resistance was calculated using the following equation[14]:

$$R_c = \left( \frac{1}{R_{sto} + R_m} + \frac{1}{R_{inc} + R_{soil}} + \frac{1}{R_{ext}} \right)^{-1} \qquad (5)$$

$R_c$ was expressed on the basis of known global radiation, surface temperature, relative humidity, land cover according to Eq. (5), using the results and assumption obtained from literature for computing and parameterization of the canopy stomatal resistance, $R_{sto}$[16,17], the mesophyll resistance, $R_m$[13,16], the canopy cuticle or external leaf resistance, $R_{ext}$ [17], the soil resistance, $R_{soil}$ [18], and the incanopy resistance, $R_{inc}$ [19], respectively.

The resistance model of the deposition velocities calculation under conditions in the Czech Republic was applied as follows: the area of the Czech Republic was divided into 37 regions, and the annual average horizontal wind velocity, $u_z$, for the years 1994 and 1998 at 37 meteorological stations was extrapolated for all these regions. The values $R_a$ and $R_b$ were calculated from the micrometeorological relations[13,15] by using the average value, $z_0$, according to individual surface types and annual average values, $u_z$, in the different regions.

Deposition velocity for particles was obtained by parametrization of friction velocity, $u_*$, for low vegetation according to Wesely et al.[20]:

$$V_d = \frac{u*}{500}, \quad L > 0 \qquad (6)$$

and for forests according to Erisman et al.[21]:

$$V_d = \frac{u*}{100}, \quad L > 0 \qquad (7)$$

The friction velocity, $u_*$, and the Monin–Obukhov length, $L$, were averaged annually.

Annual average deposition velocities, $V_d(z)$, of gases at a 10-m reference height for individual surfaces represented in areas surrounding the 37 meteorological stations on the Czech territory were calculated from Eq. (4) by averages of the values $R_a$, $R_b$, and $R_c$. The annual average deposition velocities of $SO_4^{2-}$, $NO_3^-$, and $NH_4^+$ aerosol particles were calculated using Eqs. (6) and (7). The annual average areal weighted value of deposition velocity $V_{vaz}$ was calculated for all grid cells (10 × 10 km) in the region of interest by weighing values of deposition velocities, $V_d(z)$, relating to the particular surface in a 1- × 1-km grid cell. Each grid cell (1 × 1 km) was assigned the dominant surface type.

Because monitoring stations for concentration monitoring, $C(z)$, of gaseous components and aerosol in the atmosphere are not spread evenly over the territory of the Czech Republic, the concentration data had to be interpolated to 10- × 10-km grids covering the total territory. Annual average areal weighted value of dry deposition, $D_{vaz}$, was calculated from annual average concentration, $C(z)$, annual average areal weighted deposition velocity, $V_{vaz}$, and time, $t$, in a 10- × 10-km grid resolution.

### RESULTS AND DISCUSSION

#### Wet Deposition

The annual average total wet deposition of sulphur and nitrogen compounds ($SO_4^{2-}$, $NO_3^-$, and $NH_4^+$) was estimated in the Czech Republic for 1994 at 1229 mol $H^+$ $ha^{-1}$ $a^{-1}$ and for 1998 at 1143 mol $H^+$ $ha^{-1}$ $a^{-1}$. The annual average precipitation amount, annual average wet deposition of sulphur and nitrogen compounds ($SO_4^2$, $NO_3^-$, and $NH_4^+$), and total potential acid in 1994 and in 1998 in the Czech Republic are presented in Table 1.

#### Dry Deposition

The annual average total dry deposition of sulphur and nitrogen compounds ($SO_2$, $SO_4^{2-}$, $NO_x$, $HNO_3$, $NO_3^-$, $NH_3$, and $NH_4^+$) was estimated in the Czech Republic in 1994 at 2014 mol $H^+$ $ha^{-1}$ $a^{-1}$ and in 1998 at 1582 mol $H^+$ $ha^{-1}$ $a^{-1}$. The annual average dry deposition fluxes of sulphur and nitrogen compounds and total potential acid in the Czech Republic in 1994 and in 1998 are presented in Table 2.

**TABLE 1**
**Annual Averages of Amount of Precipitation, Wet Depositions of Sulphur and Nitrogen Compounds ($SO_4^{2-}$, $NO_3^-$, and $NH_4^+$), and Total Potential Acid in the Czech Republic in 1994 and 1998 (mol $H^+$ $ha^{-1}$ $a^{-1}$)**

| Year | Annual average of amount of precipitation (mm) | Wet deposition (mol $H^+$ $ha^{-1}$ $a^{-1}$) | | | Total potential acid (mol $H^+$ $ha^{-1}$ $a^{-1}$) |
|---|---|---|---|---|---|
| | | $SO_4^{2-}$ | $NO_3^-$ | $NH_4^+$ | |
| 1994 | 688 | 506 | 317 | 406 | 1229 |
| 1998 | 759 | 454 | 328 | 361 | 1143 |

**TABLE 2**
**Annual Averages of Dry Deposition Fluxes of Sulphur and Nitrogen Compounds and Total Potential Acid in the Czech Republic in 1994 and 1998 (mol $H^+$ $ha^{-1}$ $a^{-1}$)**

| | | | Deposition (mol $H^+$ $ha^{-1}$ $a^{-1}$) | | | | | Total potential acid |
|---|---|---|---|---|---|---|---|---|
| Year | $SO_2$ | $SO_4^{2-}$ | $NO_x$ | $HNO_3$ | $NO_3^-$ | $NH_3$ | $NH_4^+$ | (mol $H^+$ $ha^{-1}$ $a^{-1}$) |
| 1994 | 790 | 88* | 407 | 86* | 162* | 424 | 57* | 2014 |
| 1998 | 485 | 88* | 343 | 86* | 162* | 361 | 57* | 1582 |

* In 1994 and 1998 data for these components were not available, therefore the values for 1993 (see Tuovinen et al.)[12] have been used. In the period 1994 to 1998 a remarkable decrease of annual average of gaseous $SO_2$ deposition can be observed.

### Total Deposition

The annual average total deposition of sulphur and nitrogen compounds on the territory of the Czech Republic has been computed as the sum of the annual averages of wet deposition and dry deposition. Spatial distribution of total deposition of $SO_x$ on a 10- × 10-km grid in 1994 and in 1998 is shown in Fig. 1 and Fig. 2, respectively. Spatial distribution of total deposition of $NO_y$ on a 10- × 10-km grid in 1994 and in 1998 is shown in Fig. 3 and Fig. 4, respectively. Spatial distribution of total deposition of $NH_x$ on a 10- × 10-km grid in 1994 and in 1998 is shown in Fig. 5 and Fig. 6, respectively. Spatial distribution of total deposition of potential acid on a 10- × 10-km grid in 1994 and in 1998 is shown in Fig. 7 and Fig. 8, respectively. Contribution

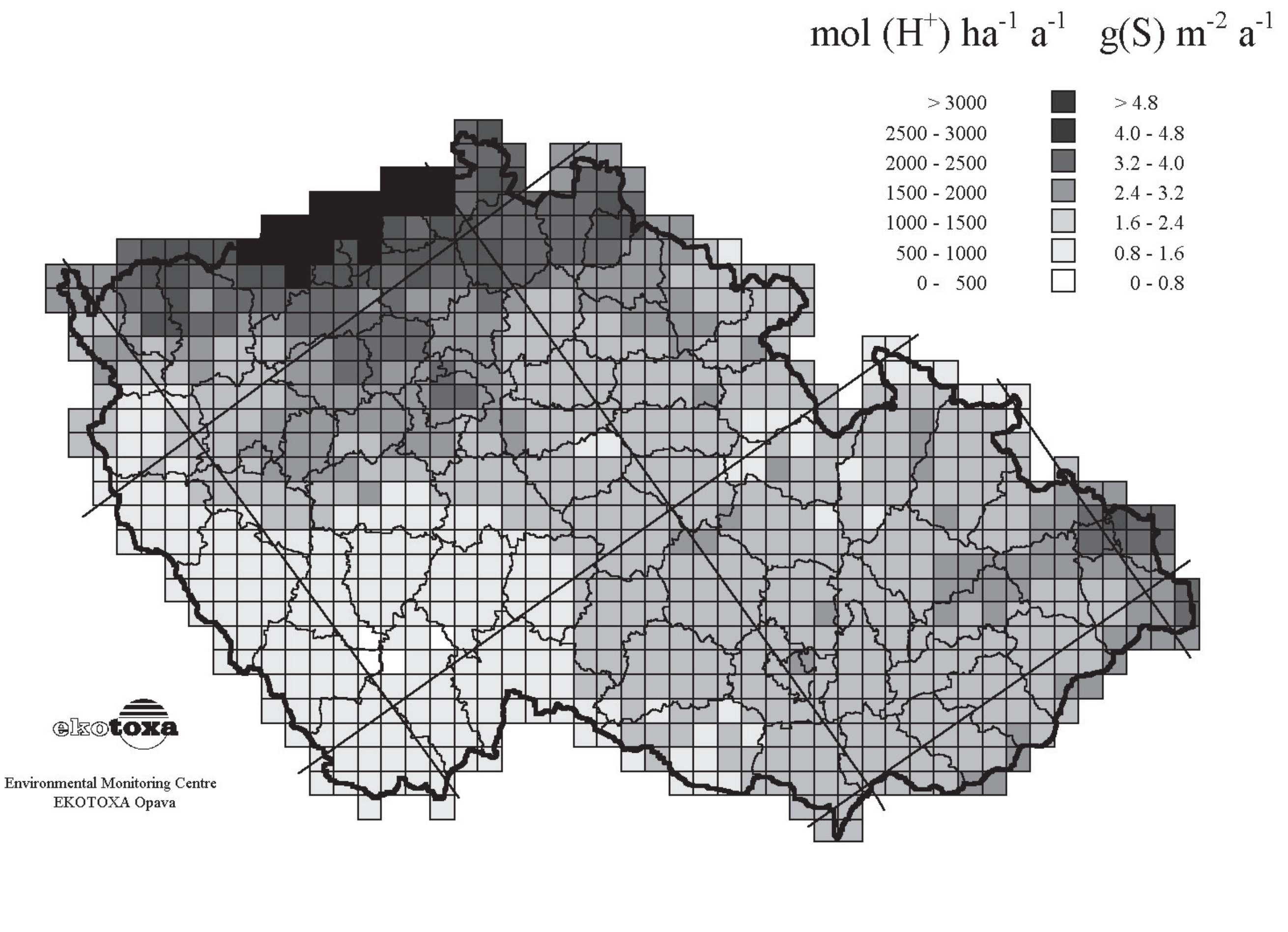

**FIGURE 1.** Total deposition of $SO_x$ in the Czech Republic on a 10- × 10-km grid in 1994 in mol ($H^+$) $ha^{-1}$ $a^{-1}$ (g(S) $m^{-2}$ $a^{-1}$).

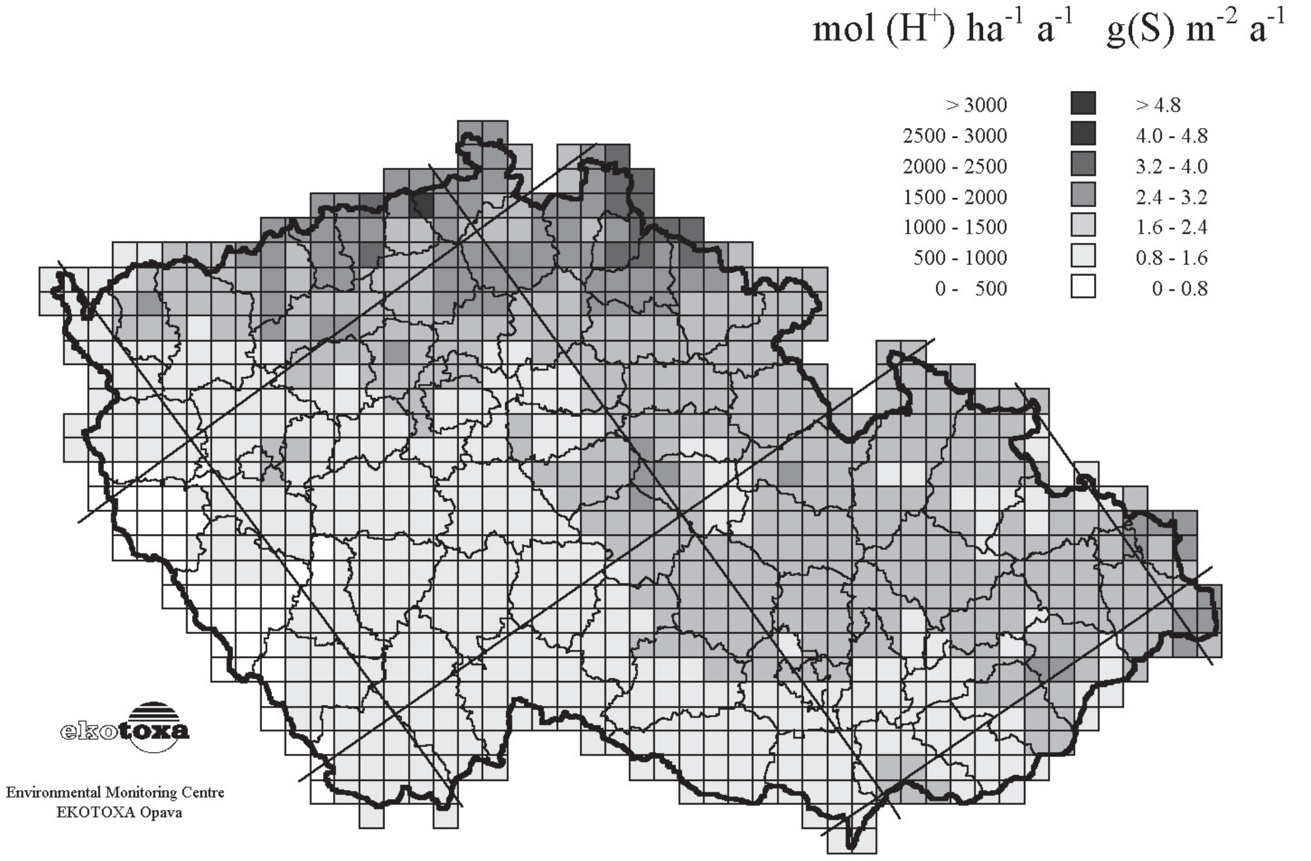

**FIGURE 2.** Total deposition of $SO_x$ in the Czech Republic on a 10- × 10-km grid in 1998 in mol ($H^+$) $ha^{-1}$ $a^{-1}$ (g(S) $m^{-2}$ $a^{-1}$).

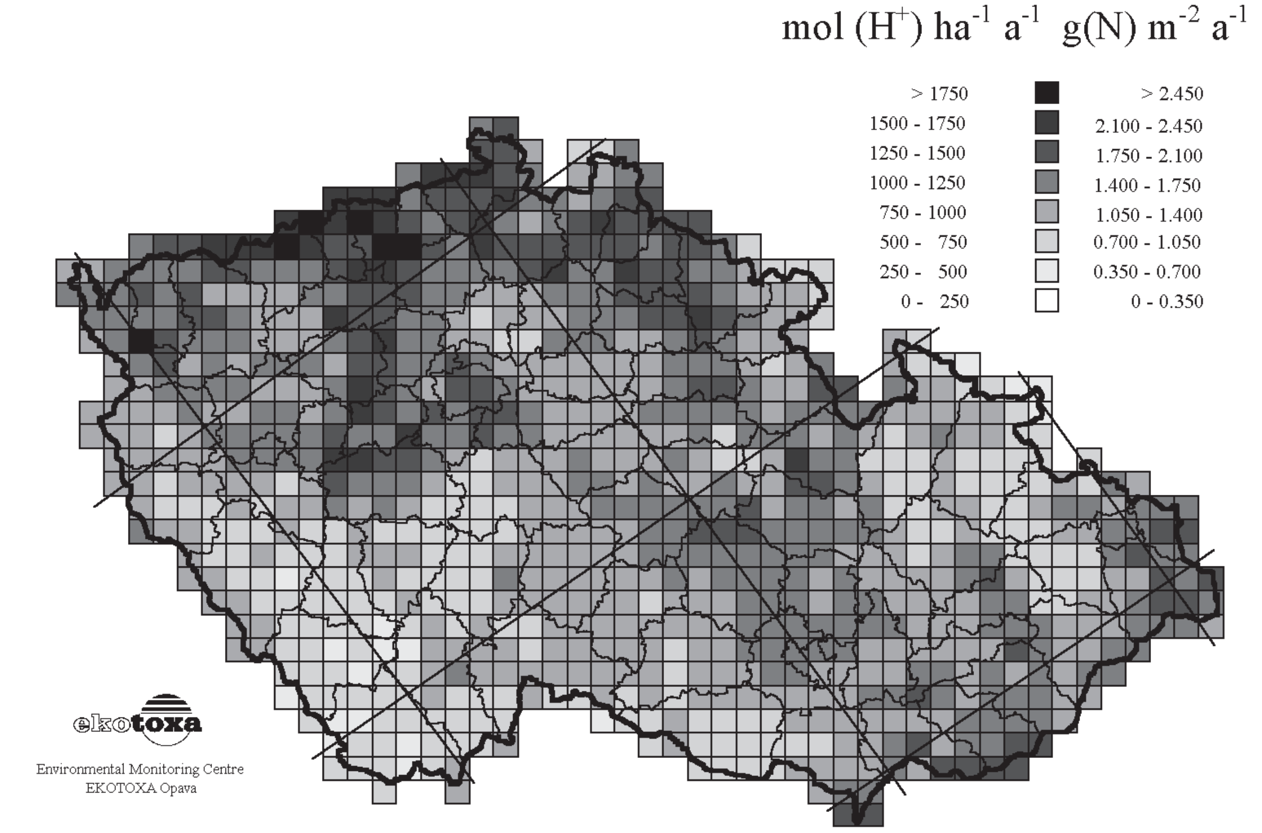

**FIGURE 3.** Total deposition of $NO_y$ in the Czech Republic on a 10- × 10-km grid in 1994 in mol ($H^+$) $ha^{-1}$ $a^{-1}$ (g(N) $m^{-2}$ $a^{-1}$).

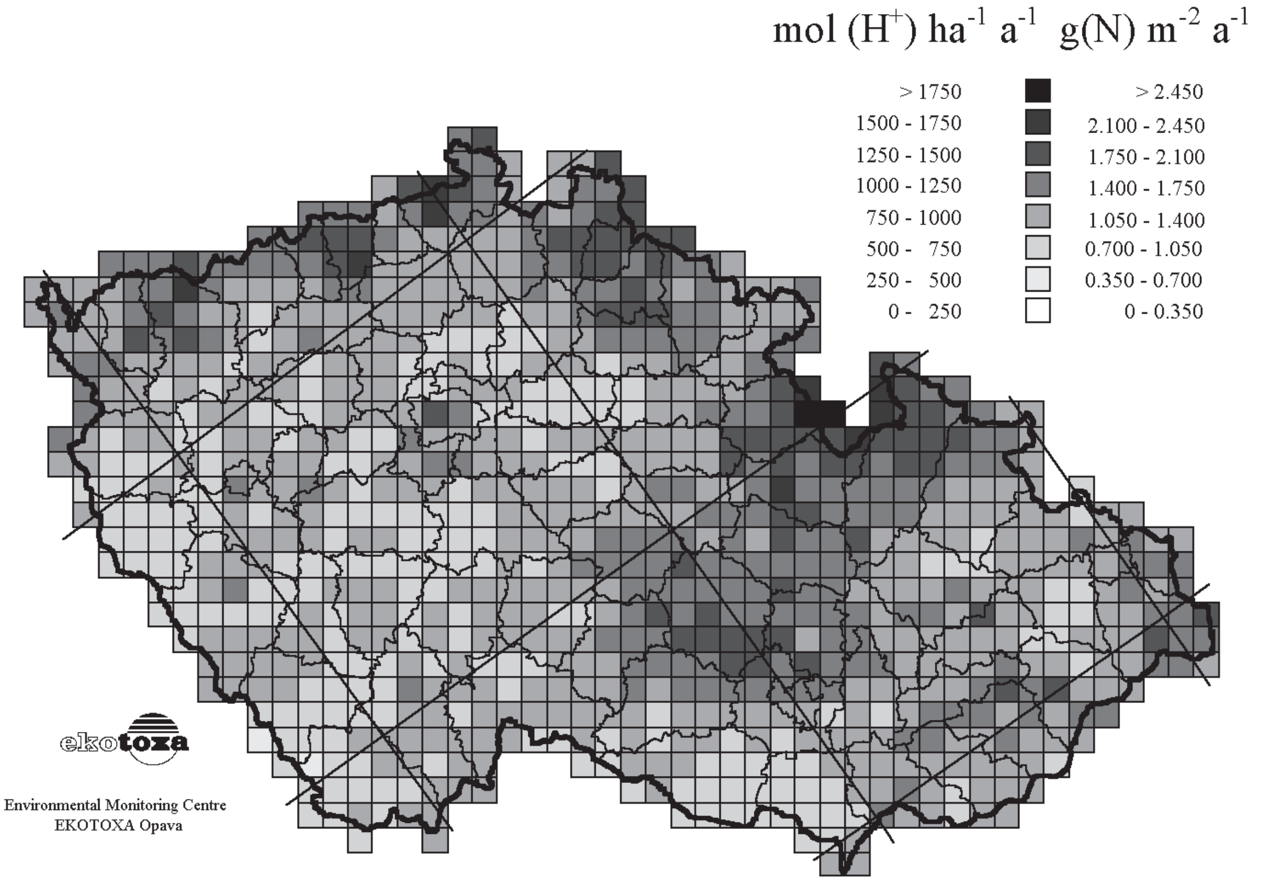

**FIGURE 4.** Total deposition of $NO_y$ in the Czech Republic on a 10- × 10-km grid in 1998 in mol ($H^+$) $ha^{-1}$ $a^{-1}$ (g(N) $m^{-2}$ $a^{-1}$).

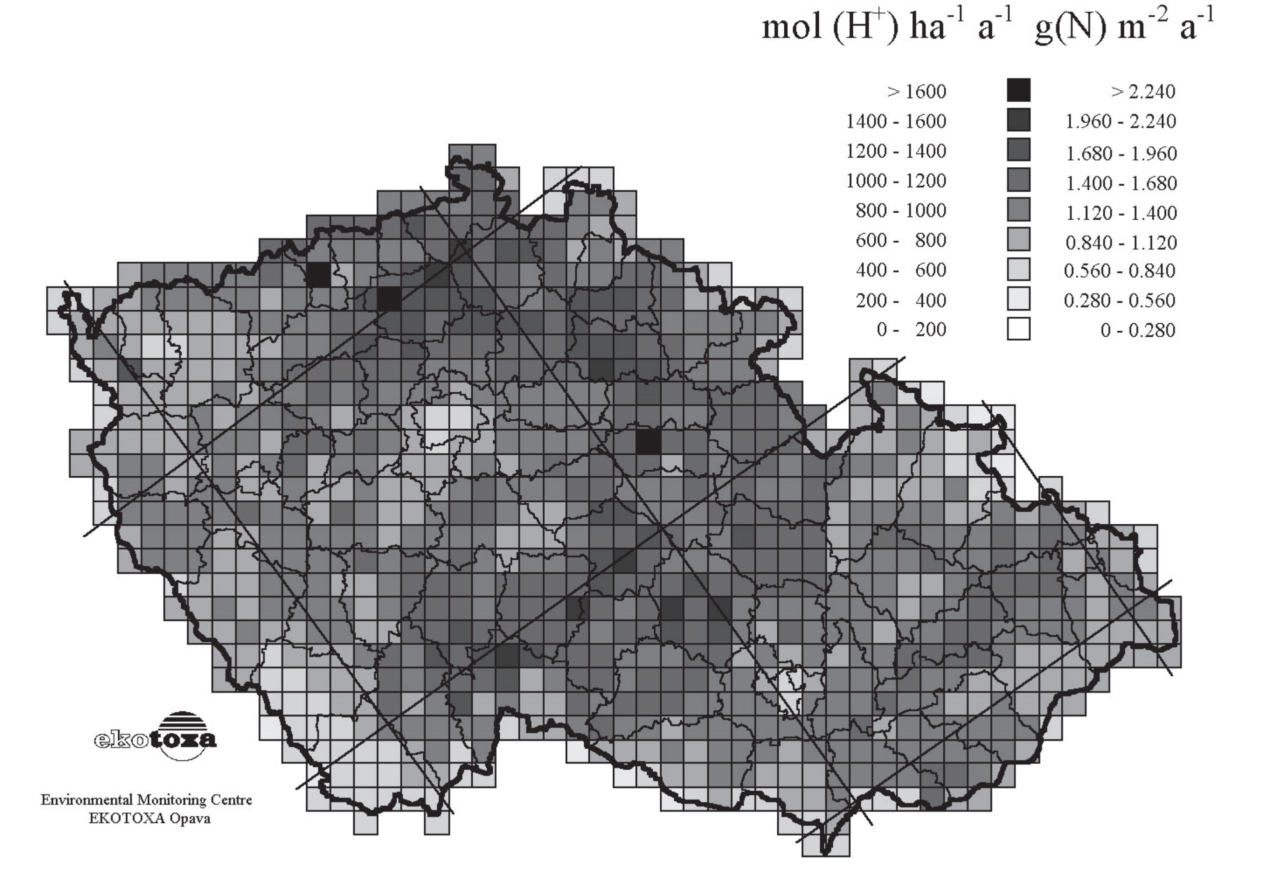

**FIGURE 5.** Total deposition of $NH_x$ in the Czech Republic on a 10- × 10-km grid in 1994 in mol ($H^+$) $ha^{-1}$ $a^{-1}$ (g(N) $m^{-2}$ $a^{-1}$).

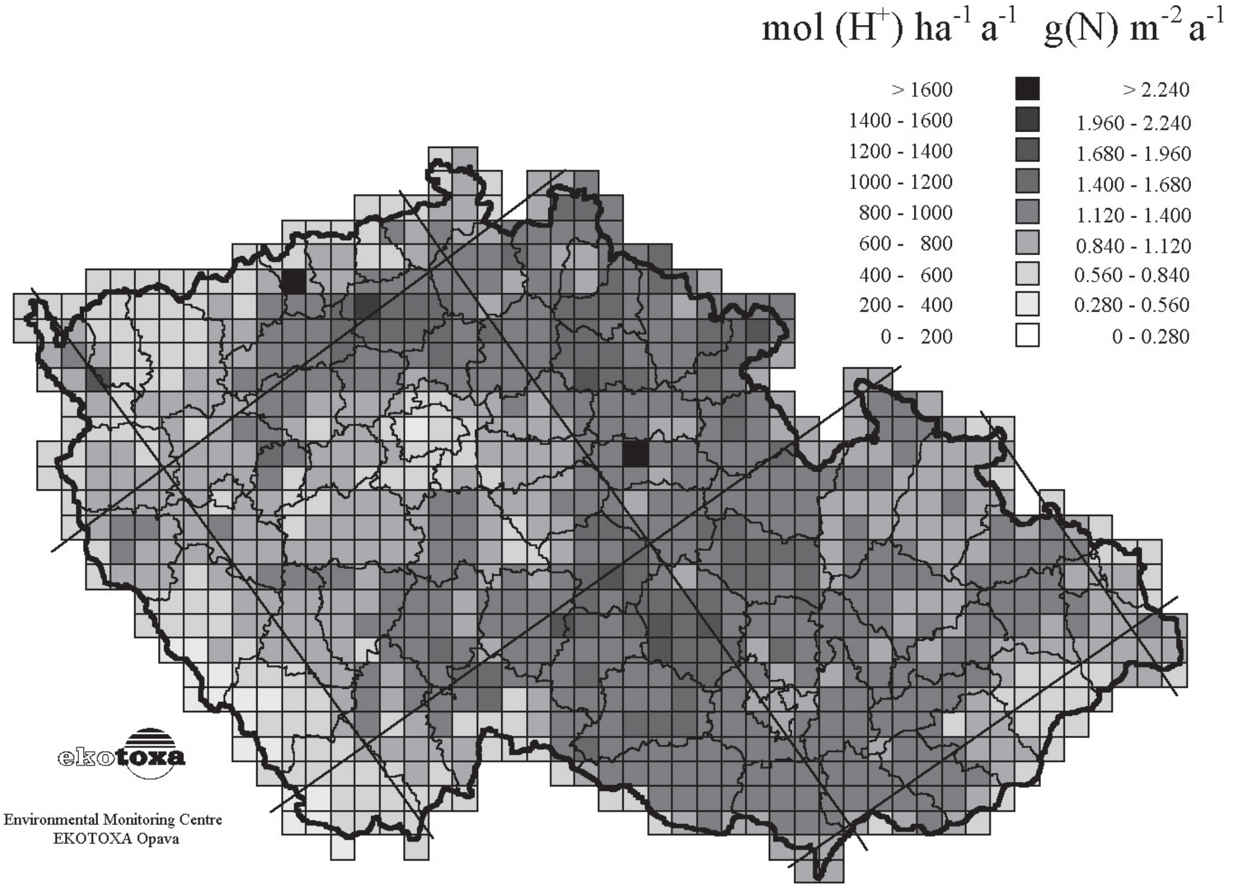

**FIGURE 6.** Total deposition of $NH_x$ in the Czech Republic on a 10- × 10-km grid in 1998 in mol ($H^+$) $ha^{-1}$ $a^{-1}$ (g(N) $m^{-2}$ $a^{-1}$).

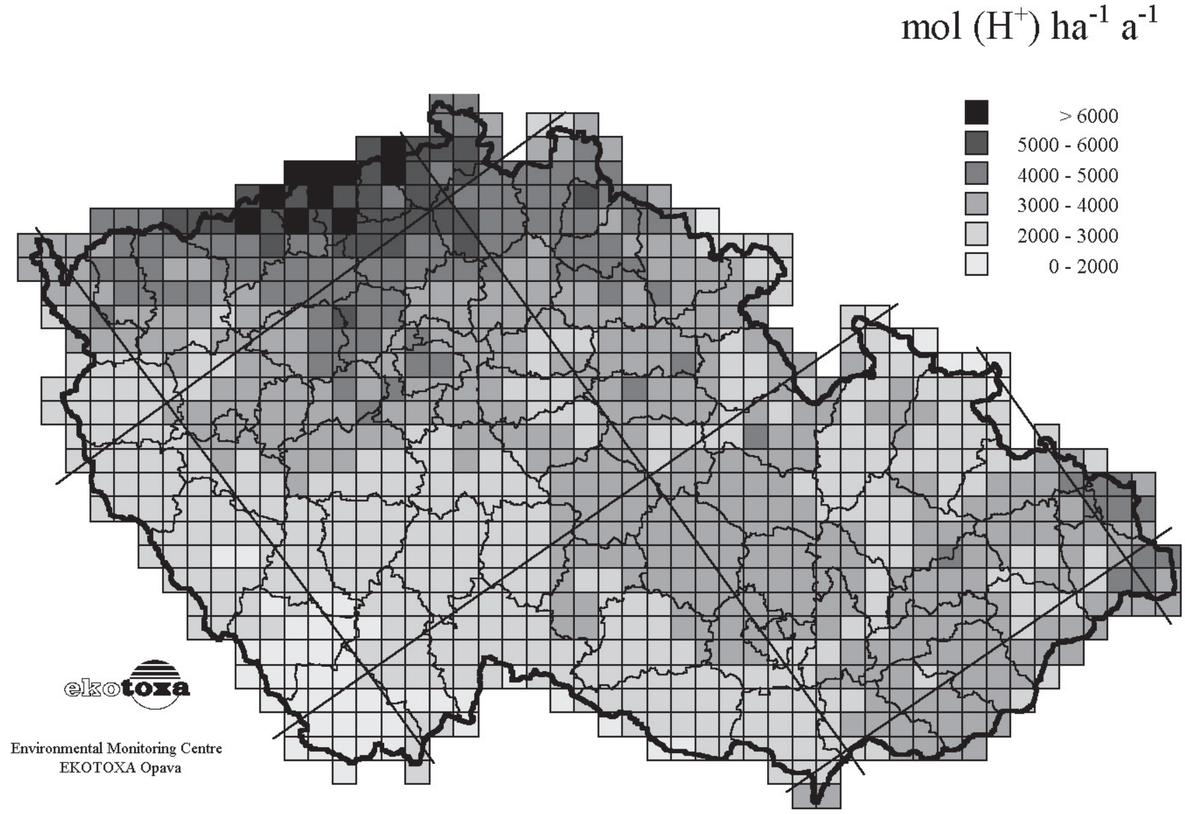

**FIGURE 7.** Total deposition of potential acid in the Czech Republic on a 10- × 10-km grid in 1994 in mol ($H^+$) $ha^{-1}$ $a^{-1}$.

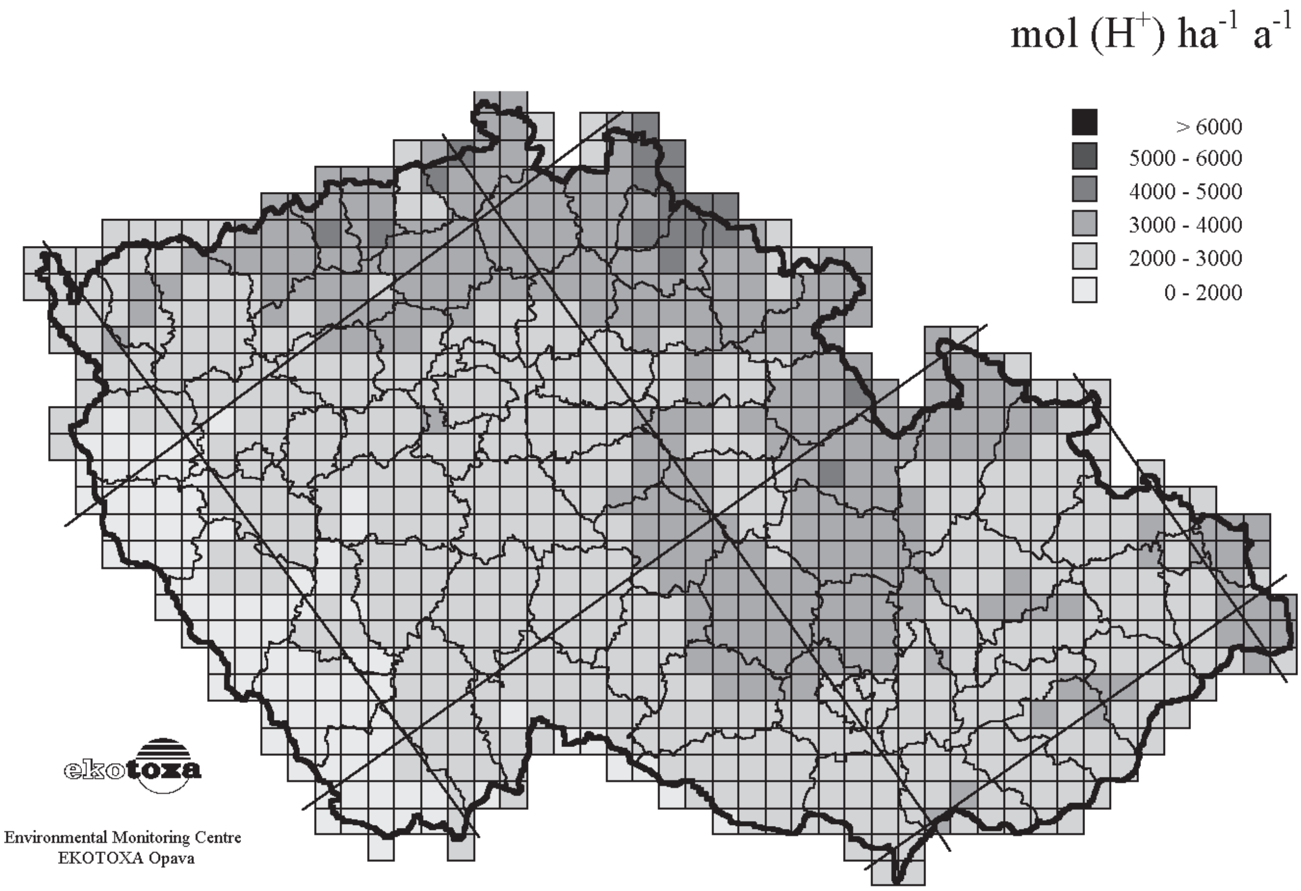

**FIGURE 8.** Total deposition of potential acid in the Czech Republic on a 10- × 10-km grid in 1998 in mol ($H^+$) $ha^{-1}$ $a^{-1}$.

(mol $H^+$ $ha^{-1}$ $a^{-1}$) of different compounds to the total potential acid deposition in the Czech Republic in 1994 and 1998 is shown in Fig. 9. Spatial distribution of the total deposition of $SO_x$ and $NO_y$ in 1994 and in 1998 shows the gradient over the Czech Republic, with the highest values in the northwest and the lowest in the south. This gradient cannot be observed for the total $NH_x$ deposition. Total deposition of $SO_x$ is influenced by the emissions from large industrial sources (the northwest part of the Czech Republic, Prague, and the Ostrava region). Total deposition of $NO_y$ is influenced by both the emissions from agglomeration areas of high combustion (industry, traffic and heating/fuel consumption) and, to lesser extent, by emissions from car traffic on important roads. The total deposition of $NH_x$ is especially influenced by $NH_3$ emission in agrarian areas with intensive animal husbandry (the north and east regions of the Czech Republic).

The dry, wet, and total depositions of $SO_x$, $NO_y$, and $NH_x$ in 1994 and in 1998 are presented in Table 3. The total potential acid deposition in 1994 and 1998 is presented in Table 4. The emission of $SO_2$, $NO_x$, and $NH_3$ in 1994 and in 1998 for comparison purposes is presented in Table 5.

Decrease of total deposition of $SO_x$ from 1994 to 1998 was caused by abatement of $SO_2$ emission in the Czech Republic (Table 5) and, to a lesser extent, in the countries of middle and western Europe. Tendency of the total deposition of $SO_x$ to decrease is especially apparent in the most loaded regions of the northwest Czech Republic, Prague, and the Ostrava region (Fig. 1 and Fig. 2). Trend in a decrease of dry deposition of $SO_x$ is much more obvious than that of wet deposition of $SO_4^{2-}$. Trend in a decrease of dry deposition of $NO_y$ is not too obvious between 1994 and 1998 (Table 3).

### CONCLUSION

The estimates derived in this study show that the annual average deposition of $SO_x$ in the Czech Republic decreased from 1384 to 1027 mol $H^+$ $ha^{-1}$ $a^{-1}$ between 1994 and 1998. The annual average $NO_y$ deposition was estimated to be 972 and 919 mol $H^+$ $ha^{-1}$ $a^{-1}$ in 1994 and 1998, respectively. The annual average $NH_x$ deposition was estimated to be 887 mol $H^+$ $ha^{-1}$ $a^{-1}$ and 779 mol $H^+$ $ha^{-1}$ $a^{-1}$ in 1994 and 1998, respectively. The annual average deposition of total (potential) acid in the Czech Republic decreased from 3243 to 2725 mol $H^+$ $ha^{-1}$ $a^{-1}$ between 1994 and 1998. $SO_x$ contributed about 43%, oxidized nitrogen species ($NO_y$) 30%, and reduced nitrogen species ($NH_x$) 27% to total potential acid deposition in the Czech Republic in 1994. Wet deposition contributed 38% to the total potential acid deposition in 1994. $SO_x$ contributed about 38%, $NO_y$ 34%, and $NH_x$ 28% to total potential acid deposition in 1998. Wet deposition contributed 42% to the total potential acid deposition in 1998.

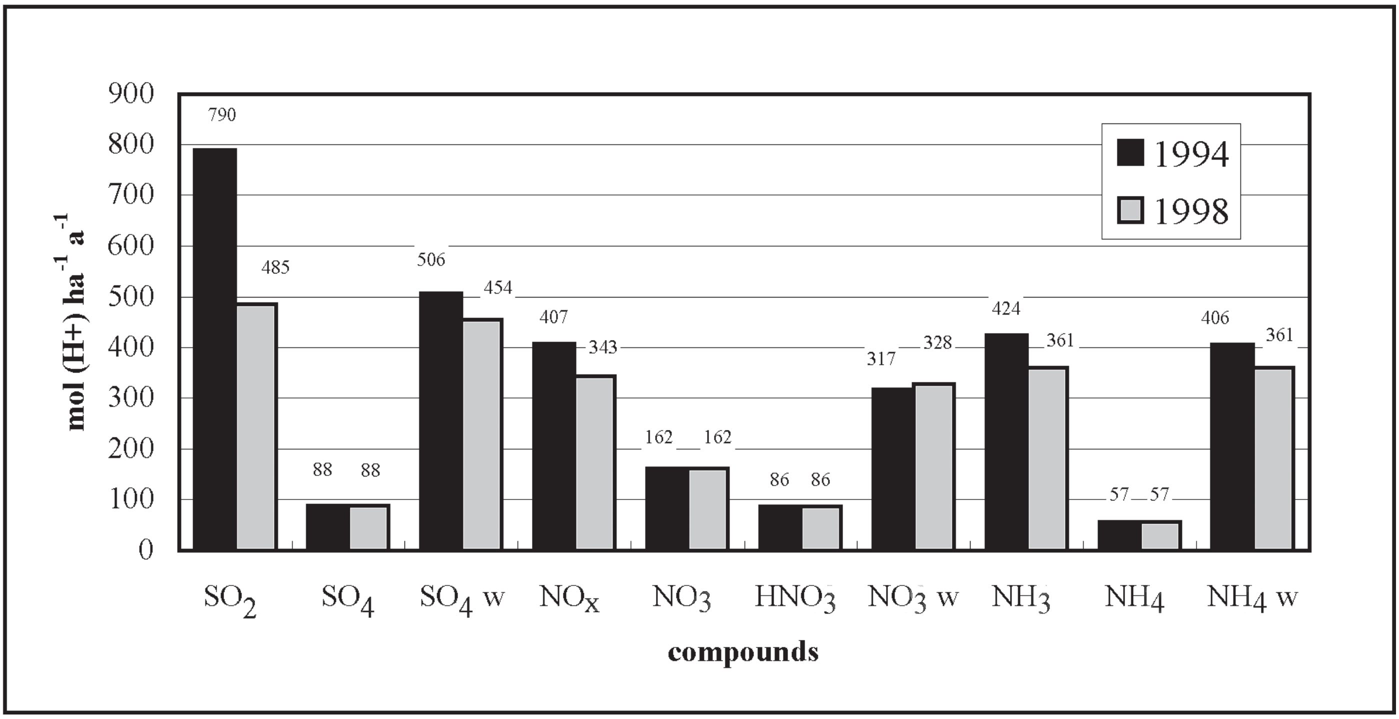

**FIGURE 9.** Contribution of different compounds to the total potential acid deposition in the Czech Republic in 1994 and 1998 in mol ($H^+$) $ha^{-1}$ $a^{-1}$ (w - wet deposition).

**TABLE 3**
**Dry, Wet, and Total Depositions of $SO_x$, $NO_y$, and $NH_x$ in 1994 and 1998 (mol $H^+$ $ha^{-1}$ $a^{-1}$)**

| | $SO_x$ (mol $H^+$ $ha^{-1}$ $a^{-1}$) | | | $NO_y$ (mol $H^+$ $ha^{-1}$ $a^{-1}$) | | | $NH_x$ (mol $H^+$ $ha^{-1}$ $a^{-1}$) | | |
|---|---|---|---|---|---|---|---|---|---|
| **Year** | **Dry** | **Wet** | **Total** | **Dry** | **Wet** | **Total** | **Dry** | **Wet** | **Total** |
| 1994 | 878 | 506 | 1384 | 655 | 317 | 972 | 481 | 406 | 887 |
| 1998 | 573 | 454 | 1027 | 591 | 328 | 919 | 418 | 361 | 779 |

**TABLE 4**
**Total Potential Acid Deposition in 1994 and 1998**
**(mol $H^+$ $ha^{-1}$ $a^{-1}$)**

| | Potential acid (mol $H^+$ $ha^{-1}$ $a^{-1}$) | | |
|---|---|---|---|
| **Year** | **Dry** | **Wet** | **Total** |
| 1994 | 2014 | 1229 | 3243 |
| 1998 | 1582 | 1143 | 2725 |

**TABLE 5**
**Emission of $SO_2$, $NO_x$, and $NH_3$ in 1994 and 1998 (mol $H^+$ $ha^{-1}$ $a^{-1}$)[10,11,22]**

| Year | $SO_2$ (mol $H^+ha^{-1}$ $a^{-1}$) | $NO_x$ (mol $H^+$ $ha^{-1}$ $a^{-1}$) | $NH_3$ (mol $H^+$ $ha^{-1}$ $a^{-1}$) |
|---|---|---|---|
| 1994 | 5032 | 1017 | 896* |
| 1998 | 1755 | 1138 | 811* |

* Calculation of emission of $NH_3$ was performed by the emission model[5,8].

The change in contributions of main compound ($SO_2$) to the total potential acid deposition in the Czech Republic in the period from 1994 to 1998 (Fig. 9) approximately reflects the change in emissions of this compound (Table 5). Decreased emissions of $SO_2$ from large industrial sources in the northwest part of the Czech Republic, Prague, and the Ostrava region between 1994 and 1998 are the main reason for decrease of total deposition of $SO_x$ (see Fig. 1 and Fig. 2) and total potential acid deposition (see Fig. 7 and Fig. 8) in these regions during this period.

### ACKNOWLEDGMENTS

This study was funded by the Ministry of the Environment of the Czech Republic. Thanks are extended to Mr. Petr Chroust for technical assistance.

## References

1. Zapletal, M. (1994) Use of geographical information systems for spatial modelling of the sulphur dioxide gas deposition on the territory of the Czech Republic. In EGIS/MARI '94 Proc. Fifth European Conf. and Exhib. Geographical Information Systems, Paris, France. Harts, J.J. Ottens, H.F., and Scholten, H.J., Eds. EGIS Foundation, Utrecht/Amsterdam, pp. 233–242.
2. Zapletal, M. (1995) Gas deposition of sulphur dioxide on the territory of the Czech Republic in 1991. In *Acid Rain Research: Do we have enough answers? Studies in Environmental Science 64.* Heij, G.J. and Erisman, J.W., Eds. . Elsevier Science B.V., Amsterdam, pp. 459–462.
3. Zapletal, M. (1996) Gas deposition of nitrogen oxides on the territory of the Czech Republic in 1994. In *Atmospheric Ammonia: Emission, Deposition, and Environmental Impacts.* Poster papers and abstracts from the International Conference on Atmospheric Ammonia, Culham, Oxford 2–4 October 1995. Sutton, M.A. Dollard, G.J., and Fowler, D., Eds. Institute of Terrestrial Ecology, Midlothian, pp.104–108.
4. Zapletal, M. (1996a) The final report of the project "Atmospheric deposition of sulphur and nitrogen on the territory of the Czech Republic in reference to the deposition limits of sulphur and nitrogen assessment and the critical loads of sulphur and nitrogen." Grant of the Ministry of the Environment of the Czech Republic PPŽP/520/4/96. Ekotoxa, Opava (in Czech).
5. Zapletal, M. (1997) Atmospheric Deposition of Acidifying Components on the Territory of the Czech Republic [Thesis]. Technical University of Mining and Metallurgy at Ostrava. Silesian University, Opava(in Czech, summary in English).
6. Skorepová, I., Roušarová, Š., Paces, T., and Zapletal, M. (1997) National Focal Center Reports: Czech Republic. In *Calculation and Mapping of Critical Thresholds in Europe — Status Report 1997.* (edited by Posch, Hettelinght, de Smet, Downing). RIVM Report No.259101007, Coordination Centre for Effects, RIVM, Bilthoven, Netherlands, pp. 66-70.
7. Zapletal, M. (1998) Atmospheric deposition of nitrogen compounds in the Czech Republic. *Environ. Pollut.* **102**, 305–311.
8. Zapletal, M., Skorepová, I., Chroust, P., And Hrivnácová, G. (2000) Multicriterial Assessment Of Negative Influence Of Air Pollution With Focus On Acidification And Eutrophication Of Natural Ecosystems Based On Critical Thresholds Principle According To Un Ece Methodology. Project Of The Ministry Of The Environment Of The Czech Republic Vav/740/4/00. Ekotoxa, Opava. (In Czech).
9. Erisman, J.W., de Leeuw, F.A.A.M., and van Aalst, R.M. (1989) Deposition of the most acidifying components in the Netherlands during the period 1980–1986. *Atmos. Environ.* **23**, 1051–1062.
10. CHMI. (1995) Air Pollution in the Czech Republic and the Chemical Composition of Precipitations. Total annual table overview 1994. Czech Hydrometeorological Institute, Prague .
11. CHMI. (1999) Air Pollution and Atmospheric Deposition in Data, the Czech Republic 1998. Czech Hydrometeorological Institute, Prague.
12. Tuovinen, J.P., Barrett, K., and Styve, H. (1994) Transboundary acidifying pollution in Europe: calculated fields and budgets 1985–1993. EMEP/MSC-W Report 1/94. Norwegian Meteorological Institute, Oslo.
13. Voldner, E.C., Barrie, L.A., and Sirois, A. (1986) A literature review of dry deposition of oxides of sulphur and nitrogen with emphasis on long-range transport modelling in North America. *Atmos. Environ.* **20**, 2101–2123.
14. Erisman, J.W. and Draaijers, G.P.J. (1995) *Atmospheric Deposition in Relation to Acidification and Eutrophication*. Elsevier Science B.V., Amsterdam.
15. Hicks, B.B., Baldocchi, D.D., Meyers, T.P., Hosker, R.P., Jr., and Matt, D.R. (1987) A preliminary multiple resistance routine for deriving dry deposition velocities from measured quantities. *Water Air Soil Pollut*. **36**, 311–330.
16. Wesely, M.L. (1989) Parametrization of surface resistances to gaseous dry deposition in regional-scale numerical models. *Atmos. Environ.* **23**, 1293-1304.
17. Baldocchi, D.D., Hicks, B.B., and Camara, P. (1987) A canopy stomatal resistance model for gaseous deposition to vegetated surfaces. *Atmos. Environ.* **21**, 91–101.
18. Meyers T.P. and Baldocchi, D.D. (1988) A comparison of models for deriving dry deposition fluxes of $O_3$ and $SO_2$ to a forest canopy. *Tellus* **40B**, 270–284.
19. van Pul, W.A.J. and Jacobs, A.F.G. (1993) The conductance of a maize crop and the underlying soil to ozone under various environmental conditions. *Bound-lay. Met.* **69**, 83–99.
20. Wesely, M.L., Cook, D.R., and Hart, R.L. (1985) Measurements and parametrization of particulate sulfur dry deposition over grass. *J. Geophys. Res*. **90**, 2131–2143.
21. Erisman, J.W. (1992) Atmospheric Deposition of Acidifying Compounds in the Netherlands [Thesis]. Utrecht University, Netherlands.
22. Barrett, K., Seland, Q., Foss, A., Mylona, S., Sandnes, H., Styve, H., Tarrassón, L. (1995) European rransboundary acidifying air pollution: ten years calculated fields and budgets to the end of the first Sulphur Protocol. EMEP/MSC-W Report 1/95. MSC-W, Oslo.

| | | | |
|---|---|---|---|
| Received: | June | 25, | 2001 |
| Revised: | June | 25, | 2001 |
| Accepted: | August | 20, | 2001 |
| Published: | September | 24, | 2001 |
